# One health pathogen surveillance demonstrated the dissemination of gut pathogens within the two coastal regions associated with intensive farming

**DOI:** 10.1186/s13099-021-00442-4

**Published:** 2021-07-23

**Authors:** Qingyao Wang, Yixiang Zhang, Qian Yang, Songzhe Fu, Baocheng Qu, Tom Defoirdt

**Affiliations:** 1 College of Marine Science and Environment, Dalian Ocean University, No. 52 Heishijiao Street, Dalian, 116023 China; 2 Key Laboratory of Environment Controlled Aquaculture (KLECA), Ministry of Education, 116023 Dalian, China; 3 CAS Center for Excellence in Molecular Plant Sciences, Shanghai Institutes for Biological Sciences (SIBS), Chinese Academy of Sciences, Shanghai, China; 4 University of Chinese Academy of Sciences, Shanghai, China; 5 Center for Microbial Ecology and Technology (CMET), Ghent University, Coupure Links 653, Gent, 9000 Belgium

**Keywords:** *Vibrio parahaemolyticus*, *Vibrio vulnificus*, Wetlands, One health, Shrimp farming

## Abstract

**Background:**

Intensive aquaculture farming has caused significant degradation of coastal wetlands and has been proposed as a reservoir for pathogenic *Vibrio* spp.

**Results:**

Gut pathogens including *Vibrio* spp., *Salmonella* spp., and *Klebsiella* spp. were isolated from bird feces, shrimp and wetland water in two typical coastal regions of China in 2015 and 2017 and were subsequently subjected to whole-genome sequencing. Meanwhile, local patient isolates were also selected to confirm the epidemiological links. Bacterial community composition analyses of the sediments that were sampled in 2015 and 2017 were conducted by the hypervariable region 4 of the *16S rRNA* gene. Together with the local clinical isolates, we observed highly related *Vibrio* isolates from waterbirds, wetlands and shrimp. Phylogenetic genome comparisons also demonstrated that sequence types ST3 and ST2414 *Vibrio parahaemolyticus* isolates obtained from aquatic animals were clonally related to patient isolates. Likewise, three *Salmonella typhimurium* isolates were also genomically related to one clinical strain. The results showed that farming activities significantly altered the community composition and resulted in the emergence of several pathogens, including *Acinetobacter*, *Mycobacterium* and *Legionella.*

**Conclusions:**

In conclusion, our results demonstrated that intensive shrimp farming in wetlands has two devastating impacts: pathogen dissemination from aquatic animals into migratory birds and transmission of foodborne pathogens into local communities.

**Supplementary Information:**

The online version contains supplementary material available at 10.1186/s13099-021-00442-4.

## Background

Destruction of animal habitats has been associated with infectious disease emergence [1–3]. For instance, the outbreaks of Ebola in humans are linked to the deforestation in West Africa [4]. Coastal wetlands are another destruction hotspot and face a range of anthropogenic threats, the most widespread and alarming of which are those posed by commercial shrimp farming [5]. Extensive loss and degradation of wetland ecosystems is also an emerging issue in China. In the recent decade, the booming of the intensive coastal farming has resulted in rapid shrinking of coastal wetlands in China. China’s coastal areas have gradually lost more than 1 million hectares in the past four decades, equivalent to 50% of the total area of coastal wetlands [6]. Due to the favorable climate and availability of space, shrimp farming has mainly developed in the tropical and subtropical coastal lowlands. In China alone, the majority of shrimp consumed are farmed in the coastal regions with an annual production of 1.76 million tons [7]. Shrimp farms require substantial quantities of water, and are primarily located alongside rivers, estuaries and coastal areas. Consequently, the rapid expansion of shrimp farming results in quick loss of coastal wetlands. For instance, Valiela et al. [8] have reported that conversion from wetland to shrimp farm is responsible for water pollution and 38% of total mangrove loss, and this is the most significant cause of mangrove loss.

Apart from wetland habitat loss and environmental pollution, another neglected issue is that as a large number of wetlands have been converted into shrimp ponds, the spillover effects of pathogens from shrimp farms into the environment and its threat to the public health have rarely been examined. Our previous study found that a widespread use of pathogen-contaminated probiotic agents in the farm would result in the spillover of pathogens such as *Bacillus cereus* from farm to environment, which might lead to human infections [9].

Bacterial enteric pathogens are responsible for a majority of diarrheal illness associated with the consumption of contaminated seafood [10, 11], of which *Vibrio* species are most widespread in estuaries and shrimp farms [12–14]. Pathogenic *Vibrio* species including O1 and non-O1 *V. cholerae*, and *V. parahaemolyticus*, have been commonly identified in shrimp farms [15, 16]. Besides, *Salmonella* spp., and *Escherichia coli* were also frequently detected in cultured shrimp and aquatic environment [17, 18]. In intensive farming systems, physico-chemical factors such as the ammonia concentration, temperature, and dissolved organic carbon levels fluctuate frequently, which may favor the growth of bacterial enteric pathogens species and result in a high abundance of pathogens in the shrimp ponds, which pose a significant health risk for the consumer [19].

Intensive shrimp farming might also accelerate the transfer of pathogens from shrimp and other aquatic animals to water birds. Recent studies revealed that wetlands, the natural food sources of water birds, are undergoing a rapid decline. With the rapid expansion of aquaculture, most of the wetlands are surrounded by a large area of intensive aquafarms in China. Zhang et al. [20] also found that the natural food for bar-tailed birds rapidly disappeared but without the decline of the number of water birds, raising the speculation that waterbirds might seek for alternative shellfish or other animals from nearby aquafarms. Our previous study further suggested that direct predation of aquatic animals was an important source of bird-carried *Vibrio* spp. [21]. Although pathogen emergence is thought to be associated with wetland conversion, clear mechanisms have not been demonstrated. One hypothesis is that edges between core and matrix wetland facilitate interspecies contact and pathogen transfer during land conversion [22]. Yet, epidemiological links between pathogens in animals and clinical samples have not been investigated. Pathogens carried by water birds and the epidemiological relationships of *Vibrio* spp. in aquatic animals have been well studied separately but have not yet been integrated [16, 21].

**Fig. 1:**
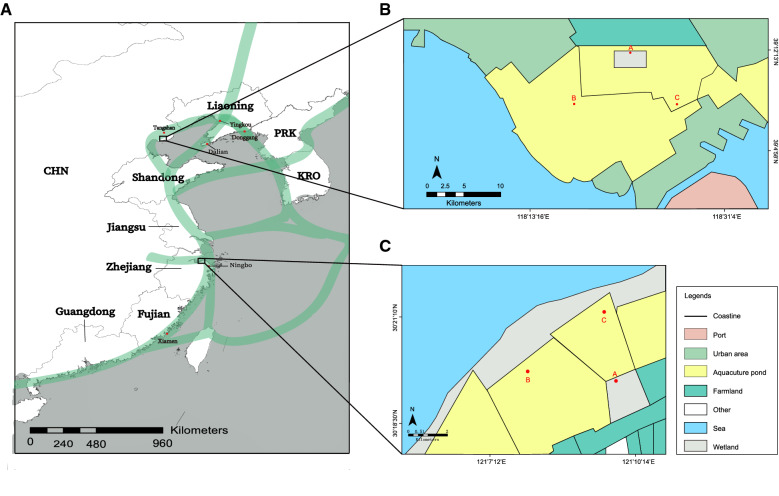
Migration route of the water birds from South China to North China (**A**) and three sampling sites in Caofeidian district (**B**) and Hangzhou Bay (**C**). Three sampling sites in each region are indicated in red. Green bands: migration route of water birds in the coastal region of China. The migratory information comes from International Union for Conservation of Nature, IUCN. The sampling sites were mapped by the ArcGIS Desktop 10.2 software (http://desktop.arcgis.com/)

To evaluate the impacts of shrimp farming on the pathogen spillover to the nearby wetlands, we selected two coastal regions. One located in the Caofeidian district (CFD) of Tangshan and the other was at Hangzhou Bay (HZB) in Ningbo, both of which are crisscrossed with shrimp farms and wetlands (Fig. 1). Gut pathogens were isolated from shrimps, wetlands and bird feces. Comparative genomic analysis was then conducted with a large dataset of sequenced genomes to determine the origin of bird-carried isolates and environmental strains. Additionally, we also analyzed the dynamics of microbial communities in the sediment of two wetlands from 2015 to 2017, which provides novel insights into the impact of aquaculture activities on the pathogen load in wetlands.

## Results

### Isolation and MLST of *Vibrio* spp. in shrimp ponds and wetlands

In 2015, five *V. parahaemolyticus* strains were isolated from the shrimp pond in the CFD, which could be subtyped into sequence types (STs), including ST415, ST1743, and ST424 (Additional file 1: Table S1). However, no pathogenic *Vibrio* spp. was isolated from water or sediment of the wetland. In 2017, two ST180 strains, one ST12 strain and one ST1800 strain were isolated from both the wetland water and shrimp pond (Table 1). Meanwhile, one *V. parahaemolyticus* strain and one *V. vulnificus* strain were retrieved from two out of 30 bird fecal samples.

**Table 1:** General features of genomes sequenced in this study

Strain	Species	Source	Location	Date	MLST	Beta-lactam	Tetracycline	Phenicol	Sulphonamide	Aminoglycoside
VP142	*V. parahaemolyticus*	Shrimp	Tangshan	Jul-15	1743	*bla*_CARB−25_	–	–	–	–
VP144	*V. parahaemolyticus*	Shrimp	Tangshan	Jul-15	1743	*bla*_CARB−25_	–	–	–	–
VP175	*V. parahaemolyticus*	Shrimp	Tangshan	Jul-15	424	*bla*_CARB−21_	–	–	–	–
VP182	*V. parahaemolyticus*	Shrimp	Tangshan	Jul-15	415	*bla*_CARB−20_	–	–	–	–
VP183	*V. parahaemolyticus*	Shrimp	Tangshan	Jul-15	1743	*bla*_CARB−25_	–	–	–	–
LH80	*V. parahaemolyticus*	Shrimp	Tangshan	Apr-17	1800	*bla*_CARB−35_	–	–	–	–
LH36	*V. parahaemolyticus*	Shrimp	Tangshan	Apr-17	12	*bla*_CARB−32_	–	–	–	–
HB12	*V. parahaemolyticus*	Shrimp	Tangshan	Apr-17	180	*bla*_CARB−29_	*tet(59)*	–	*sul2*	–
HB28	*V. parahaemolyticus*	Water	Tangshan	Apr-17	180	*bla*_CARB−29_	*tet(59)*	–	*sul2*	–
HB60	*V. harveyi*	Shrimp	Tangshan	Apr-17	/	–	–	–	*sul2*	*aph(3’’)-Ib/aph(6)-Id*
HB18	*V. parahaemolyticus*	Bird feces	Tangshan	Apr-17	180	*bla*_CARB−29_	*tet(59)*	–	*sul2*	–
HB100	*V. vulnificus*	Bird feces	Tangshan	Apr-17	/	–	–	–	–	–
NB113	*V. campbellii*	Shrimp	Ningbo	Jul-15	/	*bla*_VHH−1_	*tet(35)*	–	–	–
NB111	*V. campbellii*	Shrimp	Ningbo	Jul-15	/	*bla*_VHH−1_	*tet(35)*	–	–	–
NB118	*V. parahaemolyticus*	Water	Ningbo	Jul-15	1808	*bla*_CARB−21_	–	–	–	–
NB80	*V. parahaemolyticus*	Water	Ningbo	Apr-17	693	*bla*_CARB−33/41_	–	–	–	–
NB102	*V. parahaemolyticus*	Shrimp	Ningbo	Apr-17	1803	*bla*_CARB−21_	–	–	–	–
NB315	*V. parahaemolyticus*	Shrimp	Ningbo	Apr-17	302	*bla*_CARB−47_	–	–	–	–
NB327	*V. parahaemolyticus*	Shrimp	Ningbo	Apr-17	3	*bla*_CARB−47_	–	–	–	–
NB17	*V. tubiashii*	Water	Ningbo	Apr-17	/	–	*–*	–	–	–
NB10	*V. parahaemolyticus*	Bird feces	Ningbo	Apr-17	1	*bla*_CARB−44_	*–*	–	–	–
NB189	*V. parahaemolyticus*	Bird feces	Ningbo	Apr-17	2414	*bla*_CARB−41_	–	–	–	–
NB18	*V. tubiashii*	Bird feces	Ningbo	Apr-17	/	–	*–*	–	–	–
NB81	*V. campbellii*	Bird feces	Ningbo	Apr-17	/	*bla*_VHH−1_ */tet(35)*	–	–	*–*	*–*
NB200	*V. campbellii*	Bird feces	Ningbo	Apr-17	/	*bla*_VHH−1_ */tet(35)*	–	–	–	*–*
NB324	*V. diabolicus*	Bird feces	Ningbo	Apr-17	/	*bla*_CARB−42_	–	–	*–*	*–*
HZB82	*S. typhimurium*	Shrimp	Ningbo	Apr-17	19	–	–	–	–	–
HZB83	*S. typhimurium*	Shrimp	Ningbo	Apr-17	19	–	–	–	–	–
HZB84	*S. typhimurium*	Shrimp	Ningbo	Apr-17	19	–	–	–	–	–
HZB86	*S. typhimurium*	Stool	Ningbo	Apr-17	19	–	–	–	–	–
HB16	*Klebsiella pneumoniae*	Water	Tangshan	Apr-17	515	*bla*_SHV−145_	–	*fosA/oqxAB*	–	–
HB180	*Klebsiella quasipneumoniae*	Water	Tangshan	Apr-17	/	*bla*_OKP−B−2_	–	*fosA/oqxAB*	–	–
NXS	*Acinetobacter pittii*	Sediment	Tangshan	Apr-17	19	*bla*_OXA−335_	*tet(59)*	*mph(E)/msr(E)/floR*	*sul1/sul2*	*aac(6’)-Ib3/aph(3’’)-Ib/ aph(6)-Id*

In the HZB, no pathogenic *Vibrio* spp. were isolated from the wetland in 2015, but one *V. parahaemolyticus* strain (ST1808) and two *V. campbellii* strains were obtained from the nearby shrimp pond. In 2017, three *V. parahaemolyticus* strains were isolated from the shrimp pond, which divided into four STs (ST3, ST302, and ST1803). Meanwhile, one *V. tubiashii* strain was found in the wetland. In addition, six bird fecal samples were positive for *Vibrio* spp. in April-2017, including two *V. parahaemolyticus* strains (ST2414 and ST1), two *V. campbellii* strains, one *V. diabolicus* strain and one *V. tubiashii* strain.

### Phylogenetic analysis of *V. parahaemolyticus* confirmed pathogen circulation among birds, shrimp and humans

**Fig. 2:**
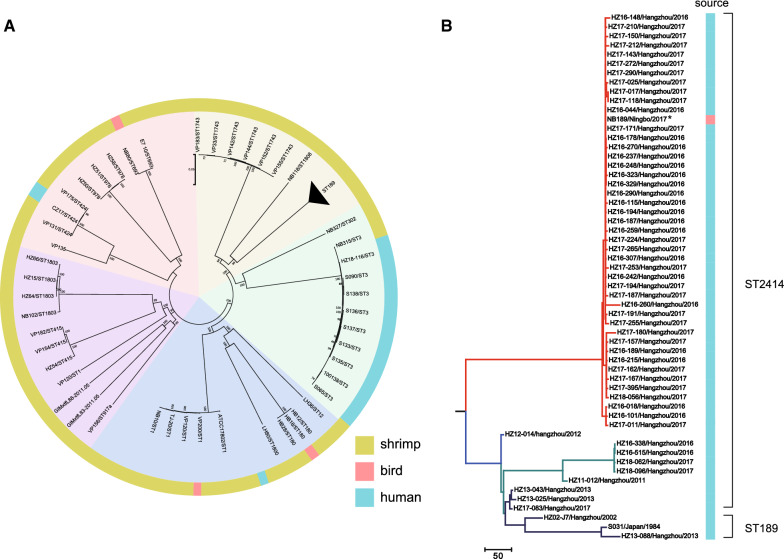
Phylogenetic tree constructed by the Maximum Likelihood method based on core genome sequences of *V. parahaemolyticus* (**A**) and Maximum parsimony phylogeny sequence type (ST) 189 and ST2414 strains (**B**); In A, the unit of the scale bar indicates the evolutionary distance in substitutions per nucleotide. In B, the unit of the scale bar indicates the number of SNPs. The number of SNP is also indicated above the branch. The bootstrap was performed with 1000 replicates

To investigate whether there is a pathogen circulation among birds, shrimp and humans, we first conducted whole genome sequencing analysis of 17 *V. parahaemolyticus* strains and compared them with 91 previously sequenced strains isolated from the two regions (Additional file 1: Table S2). Phylogenetic analysis of the 108 *V. parahaemolyticus* strains divided them into five clusters (Fig. 2A).

In our dataset, one ST180 isolate, HB18, obtained from Black-tailed Godwit *Limosa limosa*, was clustered with two isolates from shrimp in the CFD wetland with a maximum of three SNP differences (Fig. 2A). Additionally, the ST693 strain NB80 isolated from bird feces have only 14 SNP differences with strain E7_10 (isolated from fish).

Phylogenetic analysis of three ST189 and 54 ST2414 strains showed that these genomes can be divided into three clusters, the majority of which located in Cluster I, differing by less than 10 SNPs (Fig. 2B). The bird-carried ST2414 isolate NB189 was clustered with two other ST2414 strains (HZ17-143 and HZ17-171), differing by two SNPs.

Notably, one ST3 isolate NB327 was genomically identical to local ST3 strain HZ18-116 (Fig. 2B). Such epidemiological link was also observed in Tangshan; clinical isolate CZ17 has only ten SNP difference from shrimp-originated strain VP175.

### Origins of *V. campbellii*, *V. vulnificus* and *V. tubiashii* present in water birds

**Fig. 3:**
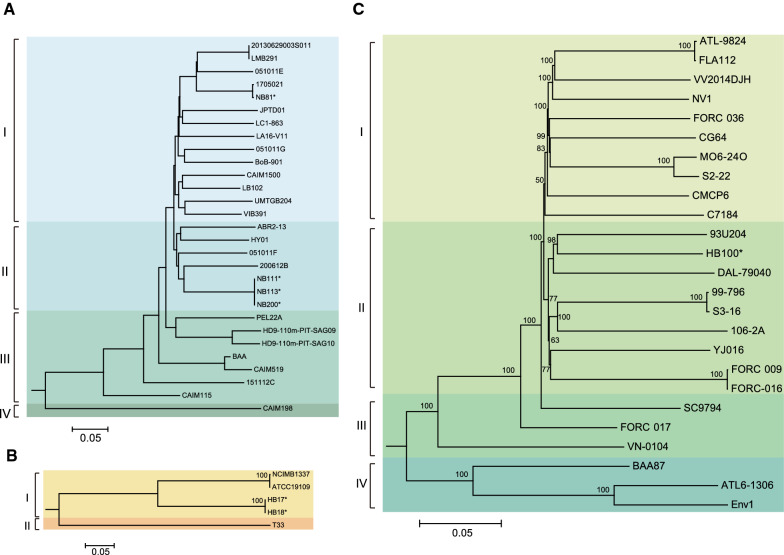
Phylogenetic relationships of *Vibrio campbellii* (**A**), *V. tubiashii* (**B**) and *V. vulnificus* genomes (**C**) based on Maximum Likelihood method. The bootstrap was performed with 1000 replicates. The numbers above the branches indicate the bootstrap value. The unit of the scale bar indicates the evolutionary distance in substitutions per nucleotide. The strains isolated in this study are indicated with asterisk sign

We subsequently determined the phylogenetic relationships of four *V. campbellii* strains isolated from this study and 28 *V. campbellii* strains from publicly available genomes. Two shrimp-carried *V. campbellii* isolates retrieved from Ningbo in 2015 were genomically identical to a bird-carried isolate, and another *V. campbellii* strain (1705021), isolated from shrimp, was clustered with bird-carried strain NB81 (Fig. 3A). In addition, together with three *V. tubiashii* strains available in public databases, five *V. tubiashii* genomes were divided into two branches, of which the bird-carried *V. tubiashii* isolate NB17 was genomically identical to isolate NB18 (from shrimp), indicating that it is possibly originated from the local shrimp (Fig. 3B).

We further conducted whole genome sequencing for one *V. vulnificus* isolate. Together with 125 publicly available *V. vulnificus* genomes (Additional file 1: Table S3), our primary analysis showed that three strains belonged to Cluster I of *V. vulnificus*, as defined by López-Pérez et al. [23]. Further genomic analysis of the Cluster I genomes revealed four lineages, of which the bird-carried *V. vulnificus* isolate HB100 was clustered with strain 93U204 and belonged to Lineage I (Fig. 3C).

### Identification and genomic epidemiology of other enteric pathogens in CFD and HZB

In 2015, no enteric pathogens were detected in CFD farming region. However, one *Klebsiella pneumoniae* isolate HB16 and one *Klebsiella quasipneumoniae* isolate HB180 were identified in the waters of 2017. In HZB, three *Salmonella typhimurium* strains, designated HZB82, HZB83, HZB84, were isolated from the shrimp in 2017, respectively. MLST showed they all belonged to ST19. *Shigella* spp., and *diarrheagenic E. coli* were not isolated in any of samples.

**Fig. 4:**
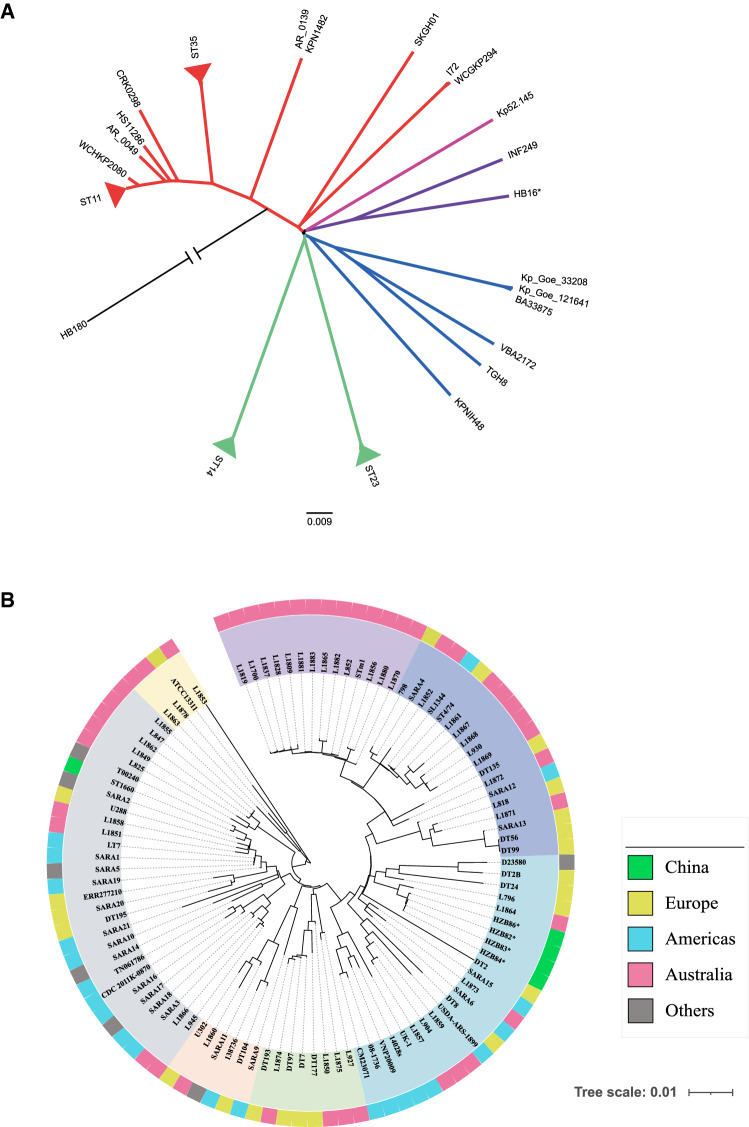
Phylogenetic tree constructed by the Maximum Likelihood method based on core genome sequences of *Klebsiella pneumoniae* (**A**) and *Salmonella typhimurium* (**B**). Genomes come from China, Europe, Americas, Australia and other countries are highlighted in green, brown, blue, pink, and gray, respectively. The unit of the scale bar indicates the evolutionary distance in substitutions per nucleotide. The bootstrap was performed with 1000 replicates. The strains isolated in this study are indicated with asterisk sign

To delineate whether *K. pneumoniae* isolate HB16 was associated with clinical samples, we additionally included 98 *K. pneumoniae* public genomes (including 16 predominant STs in China, such as ST11, ST32 and ST35) [24] and performed a phylogenetic analysis based on the whole genome SNPs. The SNP alignment of the 99 *K. pneumoniae* strains showed 170,269 SNPs. The phylogenetic analysis of *K. pneumoniae* revealed that isolate HB16 was not genomically related to any other Chinese clinical strains (Fig. 4A).

We further investigated whether three shrimp-originated *S. typhimurium* isolates were genomically related to any local clinical strains reported previously. Out of 29 local *Salmonella* strains isolated between 2016 and 2017 in Ningbo hospitals (Additional file 1: Table S4), one ST19 *S. typhimurium* isolate, designated HZB86 was picked up for whole-genome sequencing. Next, we combined 75 publicly available *S. Typhimurium* strains (Additional file 1: Table S5) with the four sequenced isolates and constructed a minimum-evolution phylogenetic tree. The results showed that HZB82, HZB83, HZB84 were clustered with the local clinical strain HZB86, with maximum difference of five SNPs, indicating that these strains belonged to the same clone with epidemiological links (Fig. 4B).

### Comparison of bacterial community composition in the sediments between 2015 and 2017 in CFD farming region

Bacterial Operational Taxonomic Units (OTUs) identified from the water in the CFD consisted of 55 phyla and 780 genera in 2015 (Additional file 1: Table S5). In 2017, bacterial OTUs constituted 51 phyla, and 630 genera. In terms of bacterial diversity, the Shannon indices decreased significantly (*p* < 0.05) (i.e., from 7.1 ± 0.2 in 2015 to 6.7 ± 0.2 in 2017) (Table 2). The number of observed species dropped from 1288 ± 44 in 2015 to 1189 ± 94 in 2017. Simpson Diversity indices decreased from 0.98 ± 0.005 in 2015 to 0.97 ± 0.004 in 2017 with a significant difference (*p* < 0.05). The Chao1 index decreased from 1457 ± 38 in 2015 to 1422 ± 206 in 2017, but the differences were not significant (*p* > 0.05).

**Table 2:** Diversity index of microbial community CFD and HZB in 2015 and 2017

Index	CFD			HZB		
	2015	2017	P value	2015	2017	P value
No. of species	1288 ± 44	1189 ± 93	0.182	1276 ± 71	1237 ± 53	0.488
Shannon	7.11 ± 0.21	6.67 ± 0.16	0.045*	5.24 ± 0.3	4.84 ± 0.3	0.178
Simpson	0.979 ± 0.0046	0.968 ± 0.004	0.035*	0.789 ± 0.015	0.854 ± 0.018	0.009**
Chao1	1457.4 ± 41.9	1422.3 ± 205.5	0.784	1023.4 ± 31	1044.4 ± 151	0.825

**Fig. 5:**
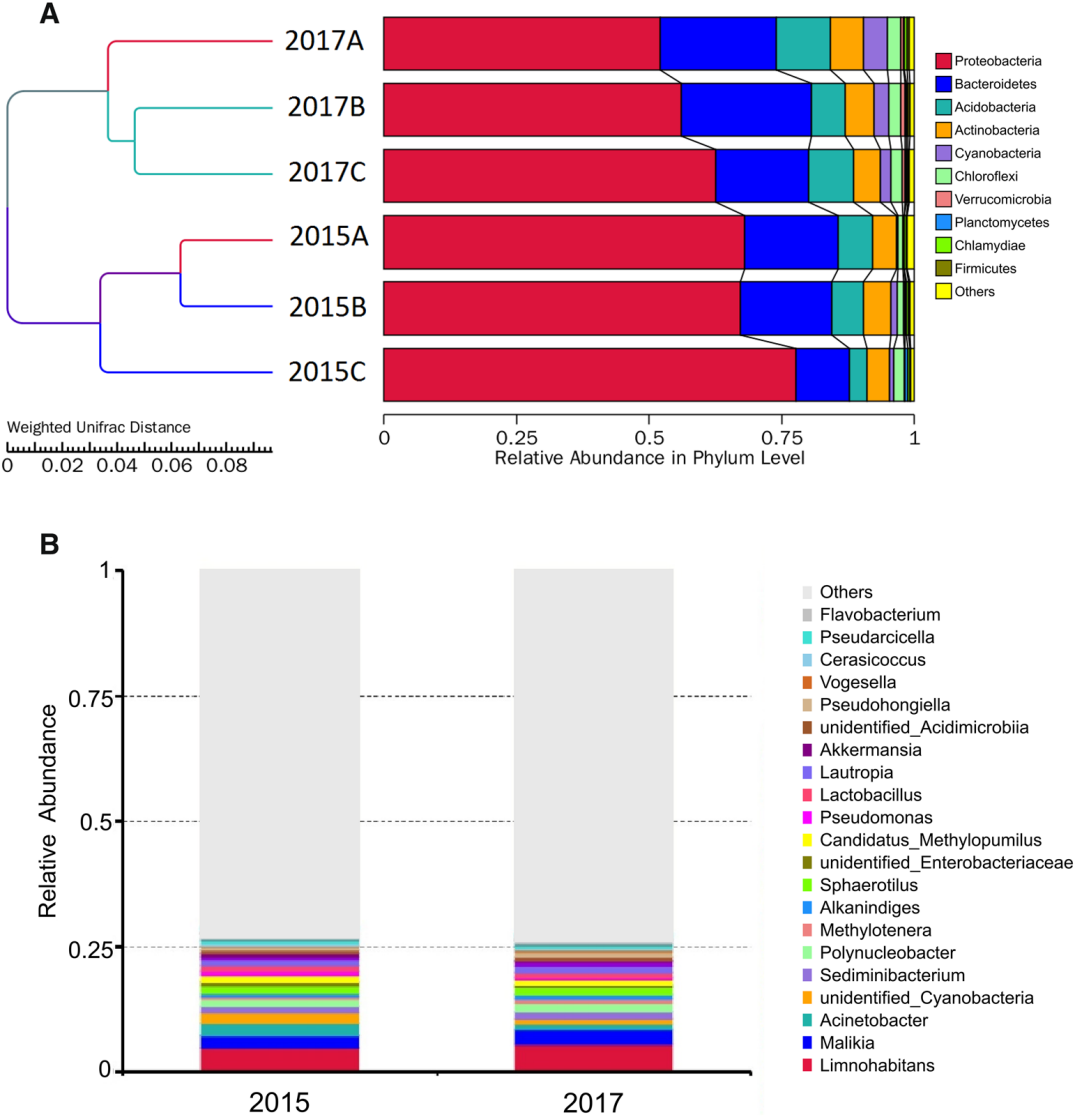
Relative abundance of bacterial communities in the sediment from shrimp pond in Caofeidian at the phylum level in 2015 and 2017 (**A**). Phylogenetic relationships and relative abundance of top 20 bacterial genus in the CFD wetland (**B**)

CFD: Caofeidian district; HZB: Hangzhou bay district; * and ** indicate significant differences at P < 0.05, and 0.01 levels, respectively,

Proteobacteria was the dominant phylum in the wetland both in 2015 and 2017 (Fig. 5A). Typical genera included *Limonohabitant*, *Actinobacteria*, *Cyanobacteria*, *Malikia*, *Candidatus Methylopumilus* and *Sediminibacterium* (Fig. 5B).

With respect to the relative abundance at genus level, there were significant differences (*p* < 0.05) at the genus level between 2015 and 2017 (Fig. 5B). The relative abundance of *Malikia* and *Limnohabitans* increased significantly (*p* < 0.05) in 2017. Meanwhile, the decrease of *Acinetobacter* and *Cyanobacteria* were observed in the sediment samples from 2017. In 2015, weighted UniFrac distance-based PCoA plots showed that the microbial composition in location A of CFD was similar to locations B and C. However, samples in location A were different in 2017, suggesting that the chemical composition of the sediment is affected by surrounding shrimp ponds, resulting in microbial composition changes.

Functional prediction analysis of the bacterial communities showed that bacterial functional groups underwent drastic changes attributed to shrimp farming activities (Additional file 2: Figure S2). Before shrimp farming, the functions of bacteria were mainly associated with aromatic compound degradation, animal parasites, sulfur reduction and chemoheterotrophy. In contrast, after shrimp farming activities have taken place, sediments had higher levels of predicted functions involved in methanol oxidation, the nitrogen cycle and carbon compound degradation. Methanol oxidation was attributed to the core OTUs affiliated with *Arthrobacter*, and methylotrophy with *Methylobacterium* OTUs, according to the FAPROTAX database. Nitrogen respiration was associated with numerous organisms, such as *Paracoccus* and *Pseudomonas*, while carbon compound degradation was associated with many bacterial species.

### Dynamics of bacterial community composition in the sediments between 2015 and 2017 in the HZB farming region

**Fig. 6:**
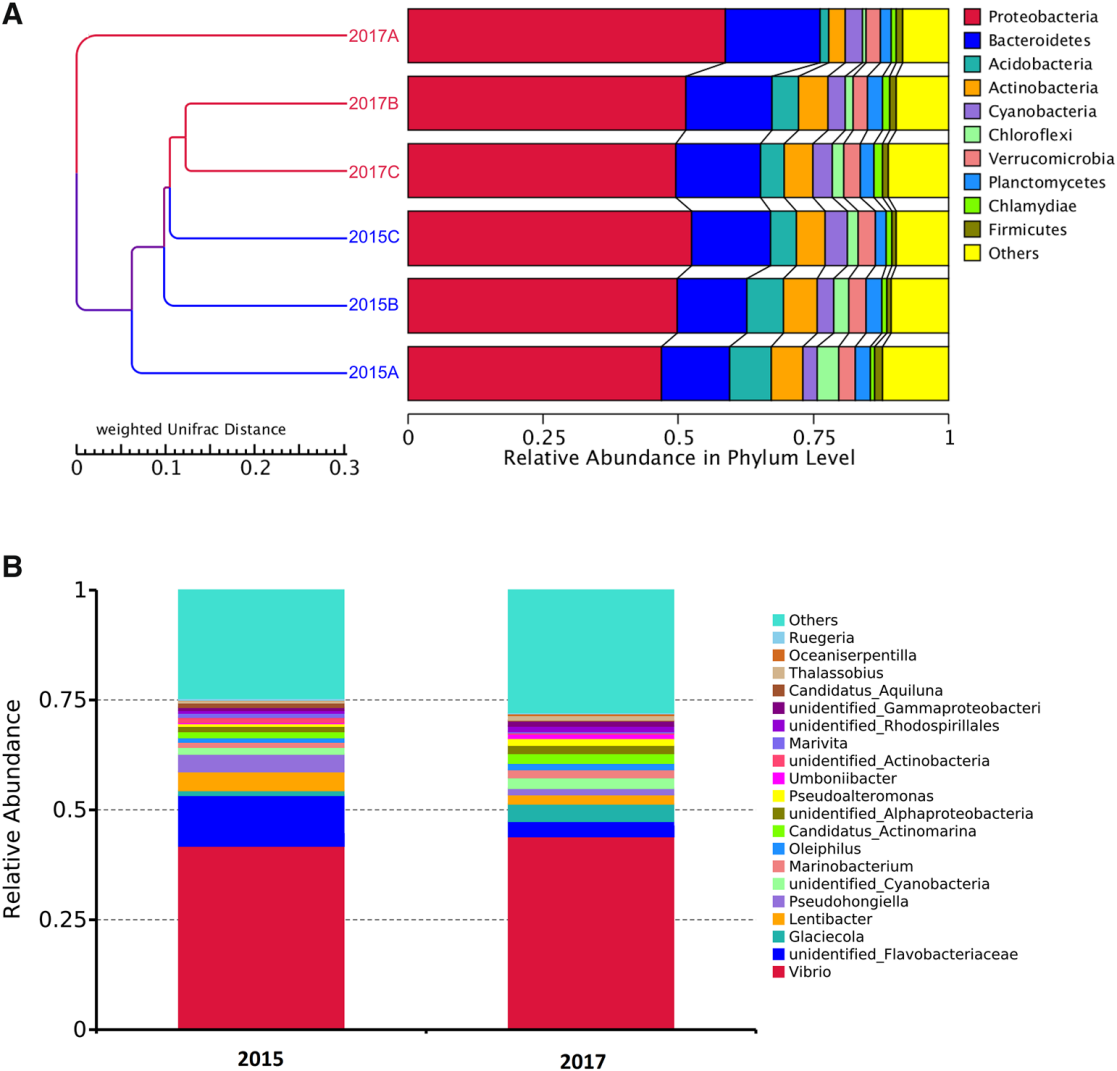
Relative abundance of bacterial communities in the sediments from shrimp ponds in Hangzhou Bay at the phylum level in 2015 and 2017 (**A**). Phylogenetic relationships and relative abundance of top 20 bacterial genus in the HZB district (**B**)

Overall, from HZB water samples in 2015 and 2017 revealed 375 and 330 genera, respectively (Table 2). Simpson index in 2017 samples is significant higher than the one from 2015 samples (*p* < 0.05). Proteobacteria was the most abundant bacterial phylum in all three sampling sites, with an average relative abundance of 75.5% ± 0.1 in 2015 and 80.9% ± 0.3 in 2017, respectively (Fig. 6A). The next two most abundant phyla were Bacteroidetes with 15.2% ± 0.5 and 7.6% ± 0.1 in 2015 and 2017, and Actinobacteria with 5.0% ± 0.5 and 3.5% ± 0.3 in 2015 and 2017, respectively. Regarding the bacterial composition at the genus level, the top three genera with the highest relative abundance were *Vibrio* with 36.3% ± 0.2, *Flavobacterium* with 7.0% ± 0.4, and *Lentibacter* with 4.3% ± 0.1 in 2015, and 45.2% ± 0.7, 2.2% ± 0.40 and 2.1% ± 0.4 in 2017, respectively (Fig. 6B). Potential bacterial pathogens including *Mycobacterium* sp., *Vibrio* sp. and *Salmonella* sp. were identified.

Functional prediction analysis of the bacterial communities revealed that there was significant difference between the samples from 2015 to 2017 (Additional file 2: Figure S3). The number of cyanobacteria and its associated function prototrophy significantly increased in 2017, indicating water eutrophication in the farming region.

In HZB, PCoA revealed that there is no significant difference among three sampling sites in 2015 (Additional file 2: Figure S1B). Intriguingly, in 2017, while the microbiota in sampling sites B and C remained comparable to the 2015 counterparts, a distinctive difference in the microbial communities could be observed between 2015 and 2017 A.

### Re-isolation of human pathogens from the sediments

Apart from successful culturing of *Vibrio* spp. and *Salmonella* spp. isolates, our metagenomics analysis had also detected the increased abundances of several potential human pathogens among the 2017 sediment samples, including *Acinetobacter* sp. and *Legionella* sp. in CFD, and *Mycobacterium* sp. in HZB. To confirm the presence of human pathogens at species level, disaggregated sediments were spread onto corresponding enrichment medium. *Acinetobacter pittii*, *Acinetobacter junii*, *Mycobacterium vaccae* and *Legionella dumoffii* were isolated from the sediments in 2017 but not in 2015. As *A. pittii* is recognized increasingly as a clinically important species, one *A. pittii* isolate NXS obtained in this study was further sequenced. Together with 125 public genomes and six previously sequenced *A. pittii* genomes (AP4, AP5, AP11, AP15, AP17 and HB181) from Liaoning provinces [9] (Additional file 1: Table S8), phylogenomic analysis revealed that *A. pittii* isolate from sediment was clonally related with another strain HB181 obtained from the ventilator of a hospital (Additional file 2: Figure S4).

### Antimicrobial resistance of the isolates

Overall, the *Vibrio* strains isolated from bird feces and aquatic animals showed similar antibiotic resistance profiles (Additional file 1: Table S1). The prevalence of antimicrobial resistance genes analyzed using ResFinder showed that all *V. parahaemolyticus* isolates carried intrinsic resistance to beta-lactams and three ST180 isolates harbored *tet*(59) and *sul2* additionally. Accordingly, a disc diffusion experiment revealed that all *V. parahaemolyticus* strains showed resistance to amoxicillin, of which ST180 isolates were also resistant to tetracycline and sulfadiazine. In addition, another bird-carried *V. diabolicus* strain harbored *bla*_CARB−42_ and was resistant to amoxicillin. Antimicrobial resistance genes were not identified in two *V. tubiashii* strains, and they also displayed no resistance to any of the tested antibiotics. Genomic analysis confirmed that all sequenced *V. campbellii* strains carried *bla*_VHH−1_ and *tet(35)*.

The *K. pneumoniae* strain HB16, which harbored *blaSHV-145/fosA/oqxAB*, showed resistance to beta-lactam, aminoglycoside, phenicol, sulphonamide, and macrolide. Genomic analysis of the *K. quasipneumoniae* strain HB180 showed the presence of *blaOKP-B-2*, *fosA* and *oqxAB* genes, which encode for beta-lactam resistance, fosfomycin resistance, and quinolone resistance, respectively.

Notably, *A. pittii* isolates NXS and HB181 were resistant to nine out of 10 drugs tested in this study (Additional file 1: Table S1). A genomic analysis showed that both strains carried 10 antimicrobial resistance genes, including *bla*_OXA−335_ (encoding for Beta-lactam resistance), *aac(6’)-Ib3*, *aph(3’’)-Ib* and *aph(6)-Id* (encoding for Aminoglycoside resistance), *floR* (Phenicol resistance), *sul1/sul2* (Sulphonamide resistance), *mph*(E) and *msr*(E) (encoding for Macrolide resistance), and *tet(59).*

## Discussion

### One health genomic surveillance demonstrated the epidemiological links between enteric pathogens in environment and communities

Although it has been well recognized that wetlands have important functions in flood conveyance, groundwater recharge, shoreline erosion control, and water storage [6], the impacts of intensive farming within wetlands on the spillover of pathogens during this process have been neglected. The CFD and HZB wetlands experienced typical human ecological problems with regard to the relationship between the ecosystem and the socio-economic system: fast aquaculture development in a non-sustainable manner for short-term economic gains. Coastal areas often face such a dilemma between economic development and environmental interests in developing countries, most of which result in a lose-lose situation.

Investigation on the dynamics of microbial communities during the human-driven land conversion is the key for illustrating pathogen spillover and seasonal epidemics [25]. For instance, in Malaysia, intensification of pig farming adjacent to bat-attracting mango plantations resulted in the emergence of Nipah virus in pig populations, due to the pig consumption of mango eaten by bats [26]. Failure to acknowledge the life-support function of mangroves was one explanation for the boom-and-bust outbreak of shrimp disease [27]. There are roughly 35,000 hectare of abandoned shrimp ponds in the Mekong Delta, of which about 50% may be rehabilitated [28]. In Thailand, 70% of previously productive ponds have been abandoned [29]. However, few studies confirmed the direct epidemiological links of enteric pathogens between agriculture environment and local communities [30]. In this study, comparative genomic analysis of *Vibrio* spp. obtained from shrimp and local clinical samples confirmed such a transmission chain. Phylogenetic genome comparisons demonstrated that ST3 and ST2414 *Vibrio parahaemolyticus* isolates obtained from aquatic animals were clonally related to patient isolates. Chen et al. [31] demonstrated that *V. parahaemolyticus* ST302 isolates were clustered with other pandemic serotype O3: K6 isolates based on enterobacterial repetitive intergenic consensus sequence PCR (ERIC-PCR), suggesting that ST302 was likely to be serovariants of ST3. However, our study confirmed that ST302 was not genomically related to ST3 as they differ by over 5000 SNPs.

Likewise, three *Salmonella Typhimurium* isolates were also genomically related to one clinical strain. These results suggested that high-density shrimp farming would cause the high prevalence of potentially enteric pathogens in farms, which might eventually result in human infections via consumption of contaminated seafood. In contrast, environmental and patient *Klebsiella* isolates were genetically distinct, suggesting that *Klebsiella* causing serious human infection did not directly originate from aquatic farms.

Sediment can be a potential reservoir of microbe due to their protective and nutrient-rich environments [32]. Thus, bacterial communities in sediments also can be used as indicators to reflect the impacts of intensive farming on pathogen spillover. Sousa et al., (2006) investigated the impacts of shrimp farming effluent on the bacterial communities in mangrove waters and found that pathogenic *Vibrio* spp. were introduced in the mangrove [33]. In this study, the investigated wetlands underwent dramatic land use changes, from a wetland to a shrimp monoculture land use system in a very short time span. Our results showed that farming activities significantly changed the bacterial community composition in the sediments, and resulted in the emergence of several pathogens. Likewise, in this study, the distinct microbial communities in the CFD farming region between 2015 and 2017 revealed the emergence and increasing of several pathogens, such as *Acinetobacter* and *Legionella*. Dynamics of bacterial community composition in the HZB farming region suggested that *Vibrio* has become a predominant genus in the sediments. Interestingly, the HZB and CFD farming regions showed a remarkably different bacterial community composition, which might be associated with the salinity, oil contamination, the land type and duration of the farming activity. The CFD farming region is far from the coast and has an average salinity of 2.3 ppt. The coastal wetland was recently converted into shrimp ponds in 2015, which explained the similarity of bacterial composition in 2015. At that time, Proteobacteria was the dominant phylum. Its sediment suffered previous oil contamination, explaining the abundance of *Alkanindiges* and other genera related to oil degradation. The abundances of several genera associated with shrimp farming were also increased in 2017, such as *Lactobacillus*, *Pseudomonas* and *Acinetobacter*.

In contrast to CFD, the HZB farming region is close to the sea shore and is strongly affected by the tide, with an average salinity of 8.5 ppt. The surrounding areas of sampling sites A, B and C were used for aquaculture activities since a decade ago. This long term shrimp farming has caused the profound alteration on the microbial community structure, resulting in similar microbiota profiles in 2015 as shown by a PcoA plot (Additional file 2: Figure S1B). Notably, the microbiota in sample 2017 A was remarkably different compared to its 2015 counterpart. The cause of the remarkable microbiota difference seen in 2017 A requires further investigation.

Field studies have shown that the conversion of coastal bulrush wetland to aquaculture pond enhanced total microbial biomass in sediment and increased the abundance of various bacterial species, many of which were human pathogens [34]. However, it has been suggested that levels of dissolved nutrients are always kept low because they are consumed by microbiota in the matured water, maintaining the stability of the microbial community and the competition between bacteria [35]. The surge of pathogenic *Vibrio* spp. in shrimp farming is mainly associated with high nutrient levels which promote the growth of fast-growing pathogens. Although it is currently unknown how the biogeochemical processes in the wetland influence the detail dynamics of bacterial community structure, our study is a first step toward understanding how the shrimp farming impacted the eco-health in the wetlands.

### Genomic analysis confirmed the dissemination of pathogenic *Vibrio* spp. among water birds and shrimp

Apart from the transmission from the farm to local communities via consumption of shrimp [16], transmission of pathogenic *Vibrio* spp. can also occur through water birds. Halpern et al. [36] suggested that the dispersal of *Vibrio* spp. by water birds might be attributable to their direct predation of chironomids and copepods. Pretzer et al. [37] revealed that bird migration explained the high genetic diversity of *V. cholerae* isolates in a European lake (Lake Neusiedl). Our previous studies also found that bird-carried *Vibrio* strains in water birds could be acquired through the direct predation of local mollusks [21]. However, very little is known about whether the conversion of wetlands to farms may accelerate the dissemination of pathogens. Our results showed that many *Vibrio* strains isolated from shrimp farms were clonally related to strains found in bird feces as well as local clinical samples. Therefore, the conversion of wetlands into aquaculture farms may result in pathogen spillover from aquatic animals to local communities and wild birds.

Using high-resolution genomic typing, we also determined the precise origins of the *Vibrio* spp. isolated from birds at two coastal farming regions in China. As a large number of wetlands have been converted to aquaculture farms, migratory birds now have to predate aquatic animals as an alternative food source. Previous field investigation also showed that in the Tangshan CFD district, the sampled shrimp and fish ponds suffered from large-scale predation by water birds, resulting in a loss of farmed animals during the bird migration season [38]. However, the high bacterial density of aquaculture ponds often leads to a high pathogen load and antibiotic resistance, and such predation behaviors are expected to enhance the acquisition of pathogens and facilitate cross-species transmission. On the other hand, altered migration routes may also facilitate contact between otherwise geographically separated host species, leading to the introductions of novel and unexpected pathogens and increasing health risks for humans. One example of this phenomenon involves outbreaks of H5N1 avian flu, which is associated with recombination that occurred within wild birds [39].

Meanwhile, the predation of water birds from aquatic farms causes another issue: the acquisition of antibiotic resistance. The overuse of antibiotics in aquaculture has been well documented in recent years, which is associated with the development of multi-drug resistance [40]. The multi-drug resistance is likely transferred by water birds along the migration route. In this study, several multi-drug resistant pathogens were isolated from the water birds inhabited stopover sites. These findings underscore that the aquacultural activity near nature reservoirs may greatly promote the spread of antibiotic resistance genes.

Owing to their long-distance movement and exposure to diverse habitats, migratory animals have far-reaching implications for the emergence and spread of infectious diseases [41]. Apart from pathogenic *Vibrio* spp. in water birds, contemporary studies have uncovered that migratory species harbor zoonotic pathogens of importance to humans, such as avian influenza viruses in water birds [42] and West Nile virus (WNV) in songbirds [43]. These studies illustrate the profound ecological and infectious consequences of migratory animals to pathogen transmission on a local scale. Therefore, restoring the food sources in wetlands is urgently needed to prevent future risks to human health.

Our study has three limitations. First, although several epidemiological links have established between animal and clinical isolates, within the limitations of sampling, we might not able to obtain all of the environmental genotypes of enteric bacterial pathogens. Thus, epidemiological links among farming region, water birds and local communities might be underestimated. Another limitation is that pathogens from other sources such as wastewater contamination from other types of food or recreational waters might also result in human infection. Thus, isolation of pathogen from other sources such as livestock would lead to better understanding on the sources of all human infections. Additional studies are required to understand whether our findings can be reproduced in other geographical areas. Thirdly, only two out of three sampling points became shrimp ponds during the study period, thus, this study lacks of sufficient sampling points to understand how the conversion of wetlands into aquaculture farms influence the detailed dynamics of bacterial community structure. Therefore, to what extent, this conversion resulted in pathogen spillover from aquatic animals to local communities and wild birds remains unknown. Nevertheless, our study is a first step towards understanding the impact of shrimp farming activities on the eco-health, highlighting an urgent need to protect the nature reserves. Future studies using metagenomic sequencing may help to determine how sustainable shrimp farming practices can improve the bacterial community structures that are variable among different wetlands.

## Conclusions

Our results clearly suggest that several problems experienced in the CFD and HZB wetlands should be urgently addressed. Comparative genomic analysis of pathogenic *Vibrio* spp. isolates obtained from wetlands, shrimp farms and local communities confirmed the dissemination of pathogens among wild birds, aquatic animals and humans. These findings confirm that pathogens can be transmitted from shrimp farms to migratory birds and the environment in estuaries, which underscores the impact of wetland destruction on the transmission of pathogens and antibiotic resistance. From a One Health perspective, the expansion of aquaculture farms near nature reservoirs must be re-evaluated. To prevent the rapid spread of pathogens, proper management actions to ensure a sustainable growth and benefit of shrimp cultivation should be adopted.

## Materials and methods

### Study area

Two coastal regions, CFD (118.46 E, 39.27 N) and HZB (121.17 E, 30.32 N) were selected for field investigation which used to be wetlands in 2015 (Fig. 1).

CFD farming region is located in the intersection of the river, the lake and the sea in Caofeidian district of Tangshan with 54,000 hectares. This region owns more than 307 types of waterbirds. There are over 50,000 waterbirds would stopover at this wetland during the spring. Since 2010, it has been surrounded by a large area of aquaculture ponds, occupying over 90% of the original wetland and were used mainly for shrimp or fish farming activities. Due to the expansion of aquaculture, many waterbirds roost in aquaculture ponds to seek food.

HZB farming region is located near the southern end of the Hangzhou Bay Bridge in the suburbs of Ningbo with 41,400 hectares. The park contains open shoals, giant reeds and wild grasses. It has vast intertidal flats (extending 3–5 km at low spring tides). The activities of water birds are strongly affected by the tide, which usually forages on the intertidal flats at low tide and rest in the high-tide roosts at high tide.

All sampled wetlands suffered from severe deterioration (Fig. 1). HZB wetland in Ningbo has been surrounded by a large area of aquaculture ponds along the coastline, which occupied more than 90% of the wetland. In 2017, field investigation showed that the core region of the wetland in Hangzhou Bay shrunk to only 300 hectares. Aquaculture ponds adjacent to the high-tide roosts are mainly used for the farming of fish and shrimp. In CFD farming region, field investigation showed that all the sampled shrimp farms located within the original wetland, of which shrimp farms and fish farms occupied 95% of water surface of the original wetland. Antibiotics, such as florfenicol, fluoroquinolone, sulfonamide, and tetracycline were widely used for the treatment of shrimp/fish disease in these farms.

### Sampling of water, shrimp and bird feces in the CFD and HZB

Two rounds of sampling were conducted in two coastal regions in July-2015 and April-2017, respectively. Sampling was selected at three angle points in wetlands, of which two points became shrimp pond in 2017 in both regions (Fig. 1). Two liters of water were collected from 1 m beneath the surface. Meanwhile, 15 sediment samples (each with 100 g) were collected both from the bottom of shrimp pond and wetland lake in two regions. Besides, 20 shrimp samples (*Penaeus vannamei*) with average individual weight of 12 ± 3.4 g were sampled from each pond.

To evaluate the pathogen spillover from shrimp to water birds, in April-2017, 30 samples of fresh bird feces were collected near the sampled shrimp ponds. Sampled bird feces came from Ruddy Shelduck (*Tadorna ferruginea*), Oriental White Stork (*Ciconia boyciana*), Common Greenshank (*Tringa nebularia*), Spoon-billed Sandpiper (*Eurynorhynchus pygmeus*), Eurasian curlew (*Numenius arquata*), Far Eastern Curlew (*Numenius madagascariensis*), and Black-tailed godwit (*Limosa limosa*). Fresh droppings were collected when only a single species presents based on field surveys. Droppings from the same species were mixed to give sufficient availability samples consisted of 10 g. All samples were placed in sterile containers and transported to the laboratory via ice package within 8 h.

### Isolation and identification of bacterial enteric pathogens from shrimp, water and bird fecal samples

Five typical bacterial enteric pathogens (pathogenic *Vibrio* spp., *Shigella* spp., *Salmonella* spp., *diarrheagenic E. coli*, and *Klebsiella* spp.) were selected for One Health surveillance.

Briefly, for pathogenic *Vibrio* spp., ten grams of sampled shrimp or bird feces were aseptically homogenized with a blender for 5 min in 100 ml alkaline peptone water (peptone, 10 g per L; sodium chloride, 10 g per L) and pre-cultured at 28 °C for 8–12 h. Subsequently, 100 µl of culture was spread on thiosulfate citrate bile salts sucrose (TCBS) agar plates (Hopebio, Qingdao, China) and incubated at 28 °C for 24 h as described previously [44]. Individual green or yellow colonies were identified and sub-cultured in tryptic soy agar plates (TSA) and incubated at 28 °C for 24 h to obtain pure isolates.

Meanwhile, the following enrichment media (Hopebio, Qingdao, China) were also used, including *Shigella* broth for *Shigiella* spp., tetrathionate broth with iodine and brilliant green for *Salmonella* spp., and mEC broth for diarrheagenic *E. coli.* Following pre-incubation at 37 °C for 48 h, 0.1 ml of bacterial suspension was plated onto MacConkey agar plate and incubated further for 24 h at 37 °C. For bacterial identification, isolates were subjected to PCR amplification of 16S rRNA genes with the bacterial universal primers (27 F/1492R) [45]. The amplicon was sequenced by ABI 3730 DNA Analyzer (Applied Biosystems, Foster City, CA) in Jingtong Biotech Co., Ltd. (Shanghai, China). The similarity of the 16S rRNA gene sequence was examined by using the BLASTn program in NCBI (https://blast.ncbi.nlm.nih.gov/Blast.cgi).

### Genome sequencing and gene content analysis

Genomic DNA was extracted from overnight cultures grown in tryptic soy broth supplemented with 1.5% NaCl (TSB15) by using bacterial genomic DNA extraction kit (TIANGEN, Beijing, China). The DNAs were indexed with a Nextera XT DNA Sample Preparation kit and sequenced on the Illumina MiSeq platform with the paired-end 2 × 300 bp protocol at Novogene (Tianjin). For the FASTQ reads, bases with a PHRED score of < 30 were removed from the trailing end by Trimmomatic (v0.36) [46]. The draft genomes were assembled *de novo* with SPAdes (v3.0) [47]. The genomes were annotated by RAST server [48]. In silico multilocus sequence typing (MLST) of *V. parahaemolyticus* and *V. vulnificus* was performed with the MLST 2.0 server (https://cge.cbs.dtu.dk//services/MLST/) at the Center for Genomic Epidemiology [49]. Antimicrobial resistance genes were identified with ResFinder [50].

### Identification of SNPs and phylogenetic inferences

For pathogenic *Vibrio* spp., the reference genomes 20130629003S011, ATCC19109, FORC_017 and RIMD2210633 were used to call the single nucleotide polymorphism (SNP) for *V. campbellii*, *V. tubiashii, V. vulnificus* and *V. parahaemolyticus*, respectively. The assemblies of *Salmonella enterica* and *Klebsiella pneumoniae* isolates were aligned to the *S*. Typhimurium LT2 genome (NC_003197) and *K. pneumoniae* genome (NC_003197) by BWA v0.7.12, respectively [51]. The SNP data used for phylogenetic analysis were identified using the SnpFilt tool [52]. The phylogenetic trees were constructed with the maximum likelihood method using RAxML 7.2.8 [53]. The generalized time reversible (GTR) with gamma rates (G) and invariant sites (I) (GTR + G + I) model was used. For closely related strains, the maximum parsimony algorithm was used to precisely identify the SNPs that differed among them, in PAUP 4.0 [54].

### Antibiotic resistance profiles

Antimicrobial susceptibility testing was carried out by using a disk diffusion assay of 10 antibiotics (Additional file 1: Table S1). The test was performed by employing a bacterial inoculum of approximately 1–2 × 10^8^ CFU·mL^− 1^ to the surface of Mueller Hinton agar plate. The plates were incubated for 24 h at 28 °C and the inhibition halo around each antibiotic disc was measured using a vernier caliper. Breakpoints of the antibiotics were interpreted using the criteria published by the Clinical and Laboratory Standards Institute [55].

### 16S rRNA gene sequencing and analysis of the sediment samples

To analyze the dynamics of microbial composition in the sediment before and after the shrimp farming in the CFD and HZB farming region, we also sampled the sediments from two shrimp ponds and one wetland lake for 16S rRNA sequencing and analysis. Sediment DNA was extracted using the Mo Bio Power soil DNA isolation kit (MO BIO Laboratories, Inc., Carlsbad, CA) following the manufacturer’s protocol. The barcoded primer set (515 F/806R) targeting the V4 hypervariable region of the 16S rRNA gene was used for PCR amplification. Subsequently, PCR products were purified using Ampure XP beads according to the manufacturer’s instructions, followed by multiplexing and sequencing on an Illumina MiSeq platform with the paired-end 2 × 300 bp protocol at Novogenes (Tianjin).

The raw fastq files were processed by UPARSE pipeline as described by [56]. Then, the operational taxonomic units (OTUs) were assigned using UPARSE at a 97% sequence identity [57]. RDP classifier v2.12 [58] and SILVA database v123 implemented in QIIME (v1.6.0) [59] were used to assign taxonomy to representative sequences of each OTU with default parameters. Functional prediction of the sediment bacterial communities was performed using Functional Annotation of Prokaryotic Taxa v.1.0 (FAPROTAX) [60]. Additionally, alpha diversities (species richness, Chao1, Shannon and Simpson indices) and beta diversities (weighted Unifrac and Bray-Curtis metrics) were analysed using QIIME [61]. Weighted UniFrac distances matrix at OTU level was calculated in R by using UniFrac function [62]. PCoA was conducted to visualize relationships of bacterial composition in the sediment before and after shrimp farming.

### Isolation of the pathogens from the sediments

Twenty-five grams of sampled sediments were aseptically disaggregated in 0.9% saline water. Subsequently, 1 ml of suspension was spread onto TSA or buffered charcoal yeast extract (BCYE) agar. Suspected *Legionella* colonies were further isolated on BCYE agar [63]. *Acinetobacter* spp. was isolated using the chromogenic HiCrome *Acinetobacter* Agar (HiMedia, India). *Mycobacterium* was isolated by Herrold’s egg yolk medium as described by Makovcova et al. [64]. PCR amplification and Sanger sequencing of the 16S rRNA gene was conducted as described above.

### Statistical analysis

Statistical analyses were conducted using R package [62]. The analysis of similarity (ANOSIM) and permutational multivariate analysis of variance (PERMANOVA) were implemented in the R package vegan to test the significance of the taxa differences between two microbial communities. The obtained profiles of KEGG pathways were further subjected to the software package Statistical Analysis of Metagenomic Profiles (STAMP) to confirm the pathway abundance differences [65]. For data following normal distribution, one-way Analysis of Variance (ANOVA) at a 5% significance level was conducted followed by the post hoc analysis Tukey Honest Significant Test.

## Supplementary Information

**Additional file 1.** Additional Tables.

**Additional file 2.** Additional Figures.

## Data Availability

The raw sequencing data were submitted to GenBank under the BioProject No. PRJNA503785.
