# Supplementary material for: One health pathogen surveillance demonstrated the dissemination of gut pathogens within the two coastal regions associated with intensive farming
Source: Gut Pathog. 2021 Jul 23;13:47. doi: 10.1186/s13099-021-00442-4 (PMC8298693; doi:10.1186/s13099-021-00442-4)

Figure S1 Principal Co-ordinates analysis (PCoA) of sediment microbiota in Tangshan (A) and Ningbo (B) based on weighted unifrac distance. In A, 2015A, 2015B, 2015C indicate samples from A, B, C sites in Caofeidian in 2015; 2017A, 2017B, 2017C indicate samples from A, B, C sites in Caofeidian in 2017. In B, 2015A, 2015B, 2015C indicate samples from A, B, C sites in Ningbo in 2015; 2017A, 2017B, 2017C indicate samples from A, B, C sites in Ningbo in 2017.


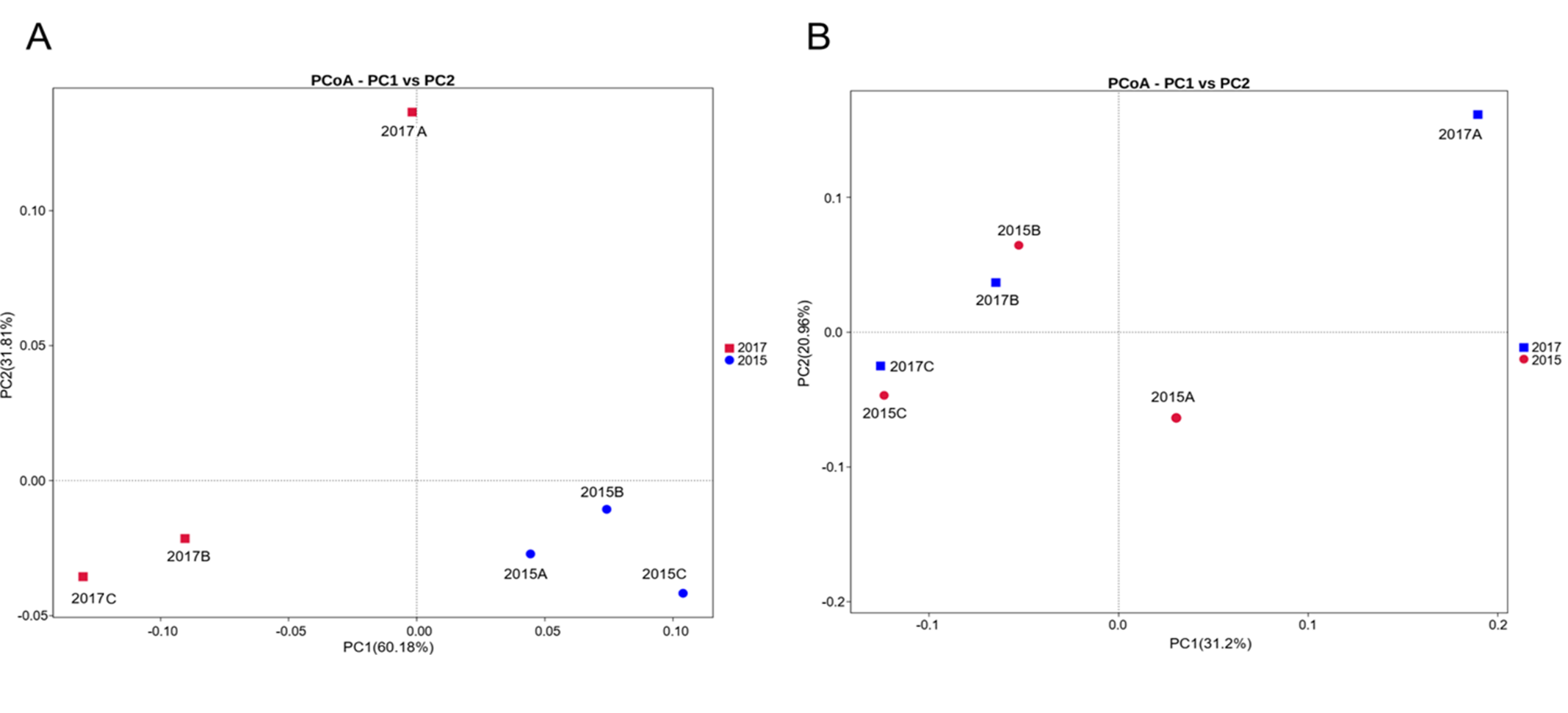


Figure S2 Functional Annotation of Prokaryotic Taxa (FAPROTAX) heatmap of bacterial communities in the Caofeidian sampling region in 2015 and 2017. FAPROTAX were used to analyse the bacterial community and potential functions during composting. Red and blue color indicate the positive and negative fold change of relative abundance of each functional bacterial group for two sampling time. Samples and categories are clustered according to Bray–Curtis dissimilarities. 15A, 15B, 15C indicate A, B, C sites in Caofeidian sampled in 2015; 17A, 17B, 17C indicate A, B, C sites in Caofeidian sampled in 2017.


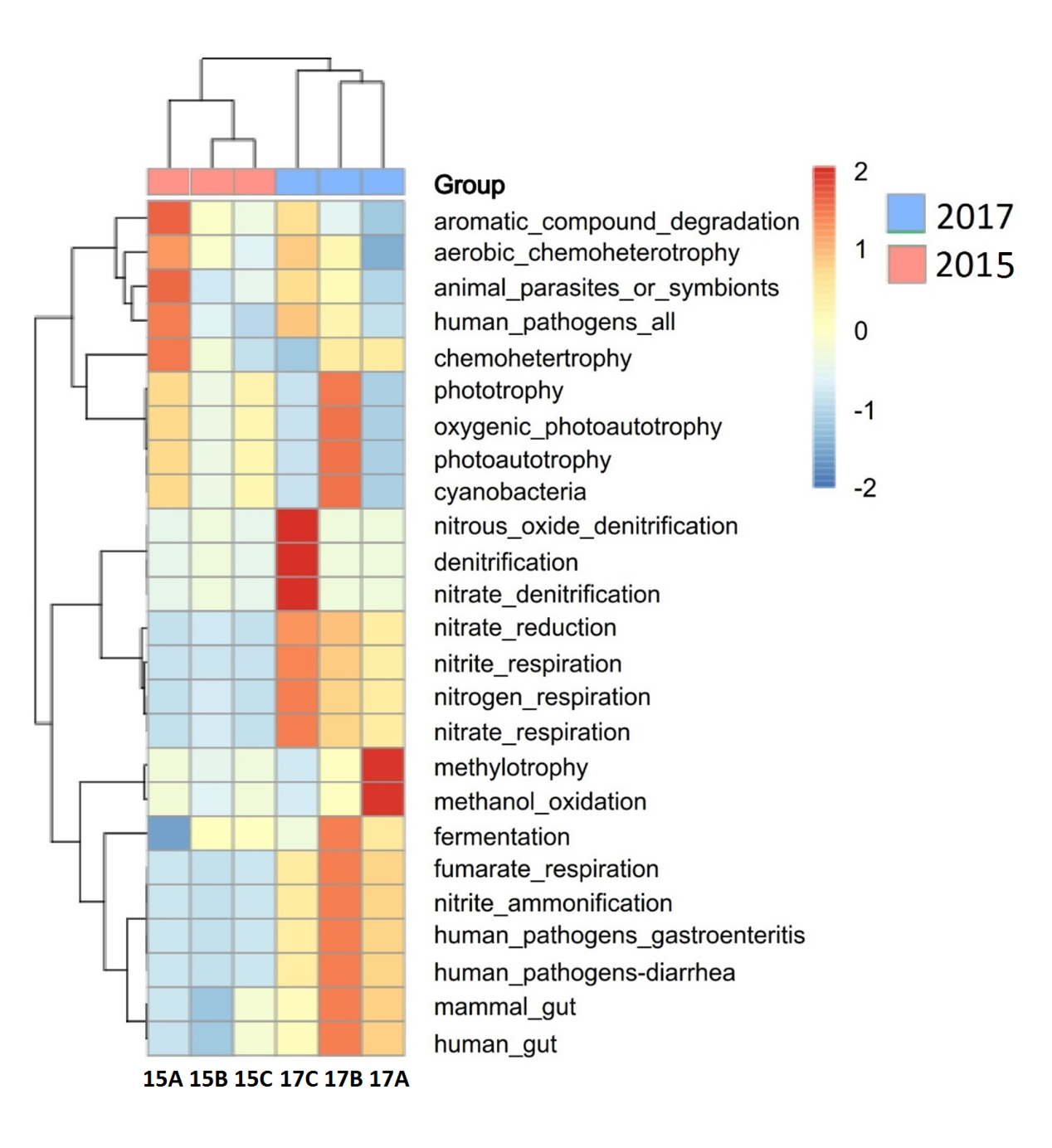


Figure S3 Functional Annotation of Prokaryotic Taxa (FAPROTAX) heatmap of bacterial communities in the Hangzhou Bay sampling region in 2015 and 2017. FAPROTAX were used to analyse the bacterial community and potential functions during composting. Red and blue color indicate the positive and negative fold change of relative abundance of each functional bacterial group for two sampling time. Samples and categories are clustered according to Bray–Curtis dissimilarities. 15A, 15B, 15C indicate samples from A, B, C sites in Ningbo in 2015; 17A, 17B, 17C indicate samples from A, B, C sites in Ningbo in 2017.


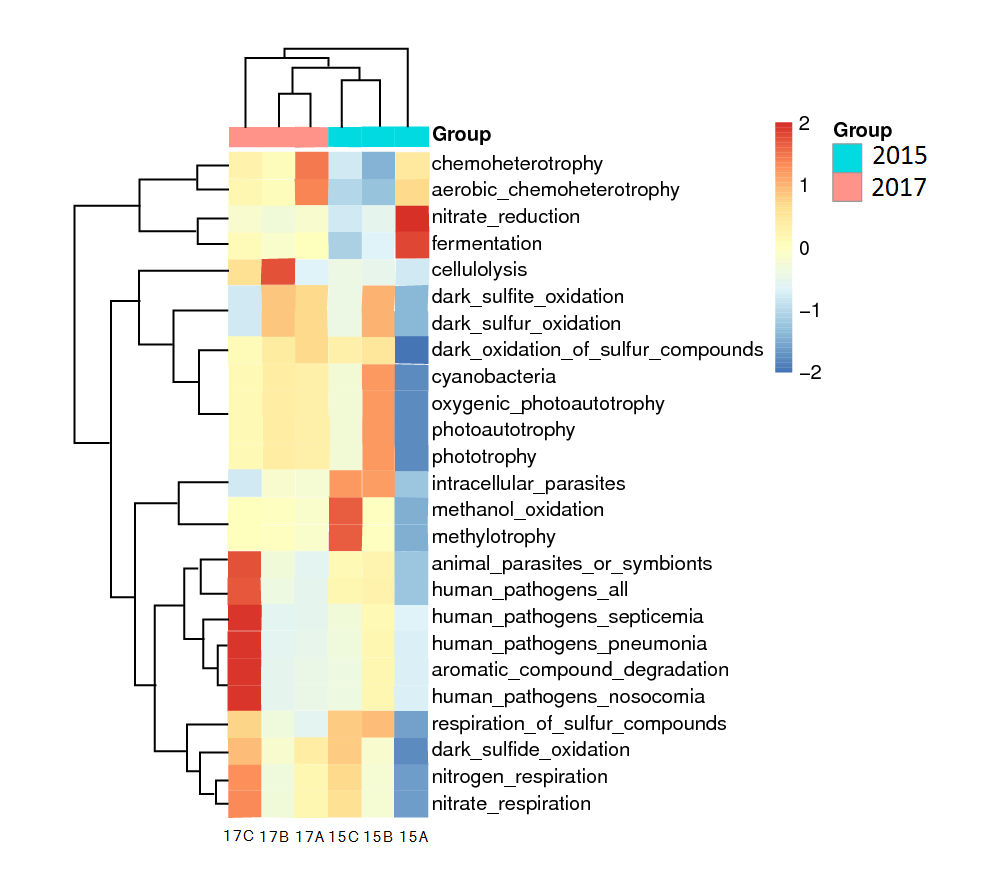


Figure S4 Phylogenetic relationships of *Acinetobacter pitti*. Phylogenetic tree constructed by the Maximum Likelihood method based on core genome sequences of *A. pitti.* The unit of the scale bar indicates the evolutionary distance in substitutions per nucleotide. The bootstrap was performed with 1000 replicates. Locations of the strains are indicated on the right side of the strain name. Strain collected in this study is indicated with asterisk sign.


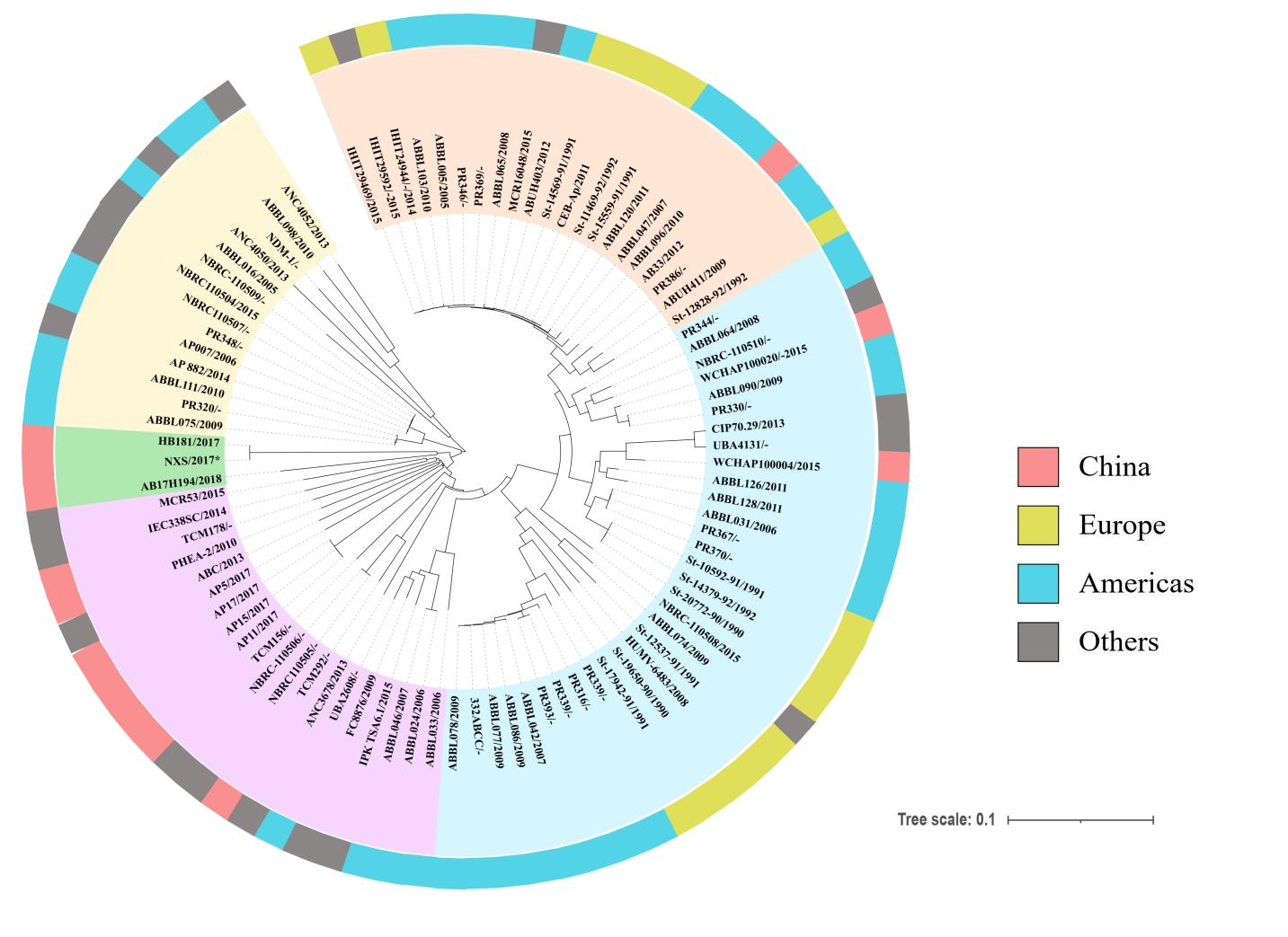

Supplement: Supplementary file 2 — Additional file 2. Additional Figures. [file 13099_2021_442_MOESM2_ESM.docx]
